# Environmental Heat Stress Decreases Sperm Motility by Disrupting the Diurnal Rhythms of Rumen Microbes and Metabolites in Hu Rams

**DOI:** 10.3390/ijms252011161

**Published:** 2024-10-17

**Authors:** Qiang-Jun Wang, Huan-Ming Yi, Jing-Yu Ou, Ru Wang, Ming-Ming Wang, Peng-Hui Wang, Xiao-Long He, Wen-Hui Tang, Jia-Hong Chen, Yang Yu, Chun-Ping Zhang, Chun-Huan Ren, Zi-Jun Zhang

**Affiliations:** 1 Anhui Provincial Key Laboratory of Conservation and Germplasm Innovation of Local Livestock, College of Animal Science and Technology, Anhui Agricultural University, Hefei 230036, China; wangqiangjun@ahau.edu.cn (Q.-J.W.); yihuanming05@163.com (H.-M.Y.); jy18027008632@163.com (J.-Y.O.); wangru3127@163.com (R.W.); wmm2314@163.com (M.-M.W.); wangpenghui15987@163.com (P.-H.W.); hexiaolong815@163.com (X.-L.H.); 18313866154@163.com (W.-H.T.); chenjiahong@ahau.edu.cn (J.-H.C.); 2 Qinghai Provincial Key Laboratory of Adaptive Management on Alpine Grassland, Qinghai Academy of Animal Science and Veterinary Medicine, Qinghai University, Xining 810016, China; greencatqi@126.com (Y.Y.); zhangchp09@126.com (C.-P.Z.)

**Keywords:** circadian rhythm, metabolome, rumen microorganism, sperm motility

## Abstract

Heat stress (HS) has become a common stressor, owing to the increasing frequency of extreme high-temperature weather triggered by global warming, which has seriously affected the reproductive capacity of important livestock such as sheep. However, little is known about whether HS reduces sperm motility by inducing circadian rhythm disorders in rumen microorganisms and metabolites in sheep. In this study, the year-round reproduction of two-year-old Hu rams was selected, and the samples were collected in May and July 2022 at average environmental temperatures between 18.71 °C and 33.58 °C, respectively. The experiment revealed that the mean temperature-humidity index was 86.34 in July, indicating that Hu rams suffered from HS. Our research revealed that HS significantly decreased sperm motility in Hu rams. Microbiome analysis further revealed that HS reshaped the composition and circadian rhythm of rumen microorganisms, leading to the circadian disruption of microorganisms that drive cortisol and testosterone synthesis. Serum indicators further confirmed that HS significantly increased the concentrations of cortisol during the daytime and decreased the testosterone concentration at the highest body temperature. Untargeted metabolomics analysis revealed that the circadian rhythm of rumen fluid metabolites in the HS group was enriched by the cortisol and steroid synthesis pathways. Moreover, HS downregulated metabolites, such as kaempferol and L-tryptophan in rumen fluid and seminal plasma, which are associated with promotion of spermatogenesis and sperm motility; furthermore, these metabolites were found to be strongly positively correlated with *Veillonellaceae_UCG_001*. Overall, this study revealed the relationship between the HS-induced circadian rhythm disruption of rumen microorganisms and metabolites and sperm motility decline. Our findings provide a new perspective for further interventions in enhancing sheep sperm motility with regard to the circadian time scale.

## 1. Introduction

With the increase in global greenhouse gas emissions, the average temperature is expected to increase by 1 °C in the next decade, possibly by 0.8–2.8 °C in 2050 and by 5 °C in 2070 [1]. In addition, global warming will lead to an increase in the frequency and duration of extreme heat events, posing a major threat to human health and sustainable livestock production [2,3]. Hu sheep are a Chinese breed reputed for their early maturity and four seasons of estrus [4]. High temperatures reduce their fertility in summer, which has caused significant economic losses to the sheep industry [5]. Rams are an important determinant of flock fertility, and the temperature of their testes is approximately 4 °C lower than their body temperature due to thermoregulation [6,7]. However, when the capacity of these thermoregulatory mechanisms is exceeded, the temperature of the testes is significantly higher in hot summers [8]. This causes oxidative stress in germ cells and leads to lipid peroxidation, apoptosis, and DNA damage in spermatogenic cells, eventually inducing the formation of abnormalities in sperm structure and a decrease in sperm motility [9,10]. Therefore, to avoid the adverse effects of HS on the reproductive function of rams, analyses of the molecular mechanisms underlying the HS-induced sperm motility decline in rams are urgently needed.

Numerous studies have revealed that HS induces spermatogenic cell damage and decreases sperm motility using static models. However, little is known about whether HS reduces sperm motility by inducing circadian rhythm disorders in rumen microorganisms and metabolites in rams. Although sheep are endotherms, HS causes an increase in core temperature and enhances its circadian fluctuation (>1 °C) [11,12]. Studies have found that almost all mammalian organs, tissues, and cells have circadian rhythm systems, and temperature is the most important zeitgeber in addition to light; previous studies, including our own, have demonstrated that a 1 °C body temperature fluctuation can reset the peripheral tissue clock [13,14,15]. These results suggest that fluctuations in body temperature amplitude caused by HS may disrupt the biological clock and lead to abnormal spermatogenesis in sheep. To further verify the role of the biological clock in the regulation of male animal reproduction, previous studies have found that a high-temperature environment changes the circadian rhythm of body temperature and serum reproductive hormones in mice and causes disorders in the biological clock and rhythmic expression of steroid synthesis genes in testicular tissue [16]. In addition, knockout of *Bmal1*, which acts as a core biological clock that regulates testosterone synthesis, reduces the expression of testicular steroidogenic genes in mice, disrupts the circadian rhythm of testosterone in serum, and reduces the level of testosterone, leading to infertility [17,18]. These results further support the hypothesis that high ambient temperatures reduce sperm motility by disrupting the biological clock in rams. However, as mice are nocturnal, their behavior, hormone secretion, and other physiological activities differ from those of diurnal animals [19]. In large diurnal animals such as sheep, whether HS reduces sperm motility by disrupting circadian rhythms is unclear.

Gut microbes regulate host growth, development, and health through metabolite signaling. In the field of reproduction, microorganisms have been found to regulate the secretion of follicle-stimulating hormone (FSH), luteinizing hormone (LH), and testosterone through their fermentation metabolites, thereby intervening in spermatogenesis [20,21]. Previous studies, including our recent work, have found that these microorganisms, which regulate various life activities of the host, are not static and remain unchanged throughout the day; rather, approximately 15% or more of the microbial species show circadian fluctuations and are affected by fluctuations in external ambient temperature and body temperature [22,23,24,25]. The external environment, such as a high-fat diet and antibiotics, can induce disorders in intestinal microbial composition and circadian rhythm, which change the expression of host biological clock genes, leading to disorders in the reproductive hormone circadian rhythm; the resulting translocation of conditioned pathogenic bacteria leads to excessive activation of the immune response, resulting in endothelial damage, destruction of the blood–testis barrier, and abnormal spermatogenesis and sperm motility [20,26]. In contrast, probiotics can effectively attenuate testicular damage caused by toxin exposure through the gut–metabolic–testicular axis [27]. However, most current studies are based on mice as a model. Whether a high-temperature environment can disrupt the rumen microbial circadian rhythm of rams and reduce sperm motility through the gut–testicular axis signal needs to be clarified in further studies.

The aim of this study is to investigate the effects of HS on sperm motility in Hu rams. We hypothesized that HS reduce sperm motility in Hu rams is associated with a disrupted circadian rhythm of rumen microorganisms and metabolites. The results of this study provide new ideas and theoretical support for mitigating the damage to sperm motility in Hu rams due to HS on a diurnal timescale.

## 2. Results

### 2.1. HS Alters the Circadian Rhythm of Body Temperature and Biochemical Indexes and Decreased Sperm Motility in Rams

**Figure 1:**
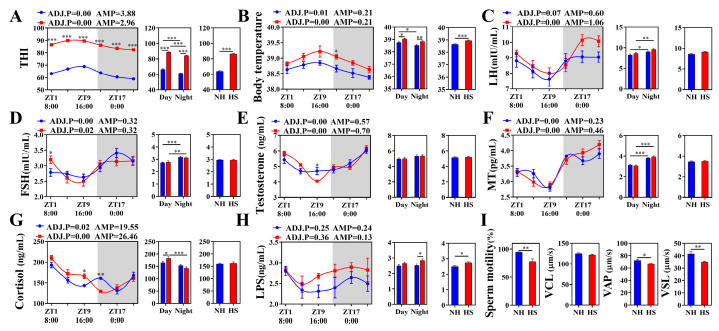
Heat stress alters the body temperature, biochemical indexes, and sperm motility in rams. (**A**–**H**) The diurnal rhythms based on a Jonckheere–Terpstra–Kendall (JTK) analysis of THI, body temperature, and biochemical indexes *(n* = 6 per time point). ADJ.P for adjusted minimal *p*-values, ADJ.P < 0.05, indicates a significant effect on circadian rhythm, AMP represents amplitude, and ZT represents Zeitgeber time. White bars in the graph represent daytime, and gray bars represent nighttime. (**I**) Sperm motility parameters (*n* = 6). Asterisks indicate significance at *p* < 0.05 (*), *p* < 0.01 (**), *p* < 0.001 (***).

The average temperature–humidity index (THI) of the heat stress (HS) group was 86.34, which was 26% higher compared to that of the non-heat stress (NH) group, indicating that the rams were in a state of HS (Figure 1A). The physiological indexes showed that HS increased (*p* < 0.05) the core body temperature and respiration rate during the daytime and nighttime (Figure 1B; Figure S1A). In addition, the cortisol concentration in the HS group increased in the daytime (*p* < 0.05), while the average level of lipopolysaccharide (LPS) in the nighttime and entire day was higher (*p* < 0.05) than that in the NH group (Figure 1C–H). The analyses of sperm motility parameters showed that HS decreased sperm motility, average path velocity, and straight-line velocity (*p* < 0.05; Figure 1I). 

### 2.2. HS Alters the Circadian Rhythm and Composition of Rumen Microbes in Rams

**Figure 2:**
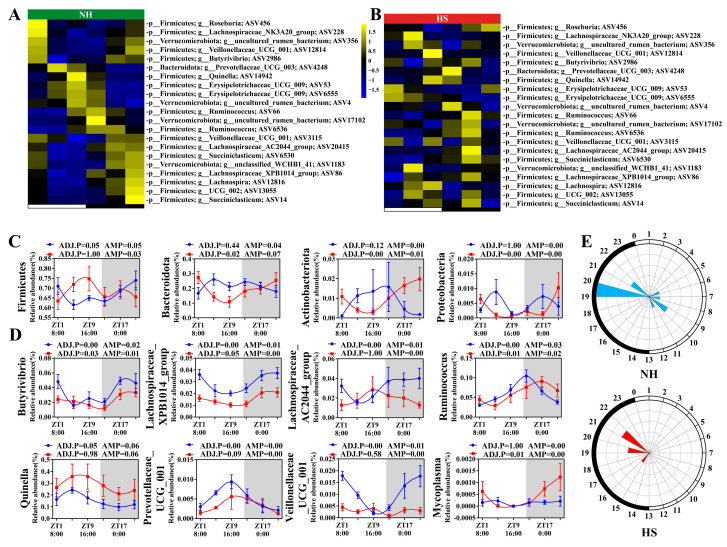
Heat stress alters the circadian rhythm of rumen microbes in rams. (**A**,**B**) Heat map showing oscillating ASVs in the NH (**left**) or HS (**right**) groups. (**C**,**D**) The diurnal rhythm of rumen microbes at phylum and genus levels (n = 6 per time point), respectively. (**E**) The polar plots represent the time when the ASV’s peak level of abundance occurred; blue (NH group) or red (HS group) shading represents the number of rhythmic ASVs with an estimated peak value for each time, as determined with JTK analysis. The radius of black concentric circles indicates the number of rhythmic ASVs, and the minimum radius of the black concentric circle represents one ASV. The black arc on the left side of the polar plot indicates the day/night cycle.

Our results showed that rhythmic (ADJ.P < 0.05) amplicon sequence variants (ASVs) in the NH group mostly belonged to Bacteroidetes and Firmicutes; these ASVs were associated with a loss of circadian rhythm in the HS group (Figure 2A,B). Analyses at the phylum level showed that Firmicutes exhibited circadian rhythms only in the NH group (ADJ.P < 0.05), whereas Bacteroidetes, Actinobacteria, and Proteobacteria exhibited circadian rhythms only in the HS group (Figure 2C and Figure S1B). Similarly, the analysis at the genus level revealed that 12.87% and 0.04% of the microorganisms in the NH and HS groups exhibited circadian rhythms (ADJ.P < 0.05), respectively (Figure S2A). Among these genera, *Lachnospiraceae_AC2044 _group*, *Quinella*, and *Prevotellaceae_UCG_001* exhibited circadian rhythms (ADJ.P < 0.05) only in the NH group, *Mycoplasma* exhibited circadian rhythms only in the HS group (Figure 2D), and the NH group of rhythmic microbial abundance peaks occurred during the daytime and nighttime, while the HS group of rhythmic microbial abundance peaks occurred only at nighttime (Figure 2E). In addition, 11.98% and 4.19% of microorganisms in the NH and HS groups showed circadian rhythms (ADJ.P < 0.05) at the species level, respectively (Figure S2A). Similarly, the polar plot and heat map showed that the peak value of the NH group appeared during the daytime and nighttime, whereas that of the HS group was distributed during the nighttime (Figure S2B–D). Moreover, compared with the circadian rhythm-related microorganisms in the NH group, the circadian rhythm-related microorganisms in the HS group belonged to Actinobacteria (Figure S2C,D).

To further investigate whether HS alters the rumen microbial structure in rams, OPLS-DA analyses indicated that HS significantly affected the rumen microbial structure (Figure S2E). According to the analysis of similarity (ANOSIM), differences (*p* = 0.001; r = 0.107) in rumen microbial structures occurred between the NH and NS groups (Figure S2F). Similarly, to investigate whether HS alters the rumen’s microbial composition at the genus and species levels in sheep, LEfSe and rank sum test analyses found that the NH group was enriched in *Lachnospiraceae* spp., *Veillonellaceae* spp., and *Prevotellaceae_UCG_001*, whereas *Quinella*, *Pseudoscardovia*, and *Lachnobacterium_bovis* were enriched in the HS group (Figure S2G). 

### 2.3. HS Alters the Diurnal Pattern of Rumen Metabolites in Rams

**Figure 3:**
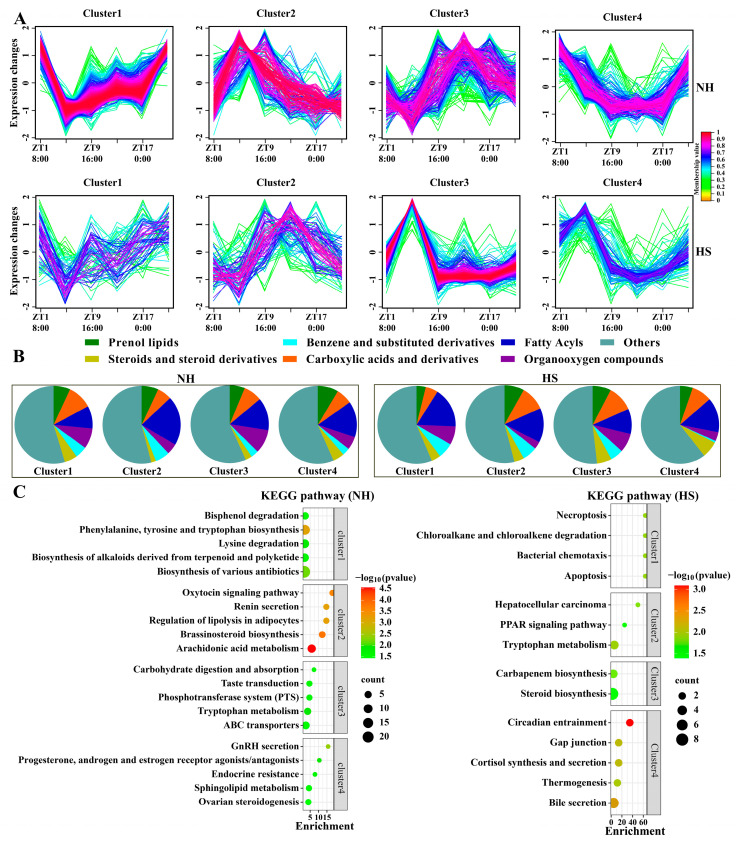
Heat stress alters the diurnal pattern of rumen metabolites in rams. (**A**) Identification of distinct temporal patterns of metabolites in the NH and HS groups using fuzzy c-means clustering. The y-axis represents normalized data based on all metabolites within each cluster. (**B**) Classification of metabolites. (**C**) KEGG analysis (*p* < 0.05) of the metabolites within each cluster in the NH and HS groups.

Environment-induced changes in microbial circadian rhythms regulate host metabolic processes. The JTK analysis of the 3439 metabolites identified in rumen fluid showed that 70.30% and 24.48% of the metabolites in the NH and HS groups showed circadian rhythms (ADJ.P < 0.05), respectively, and 793 metabolites showed circadian rhythms in both groups (Figure S3A). The polar plots and heat map showed that the abundance of rhythmic metabolites in the NH group peaked mainly during the daytime, whereas that in the HS group was distributed throughout the day (Figure S3B–D). To further elucidate the regulatory role of rhythmic metabolites in physiological processes in rams, a fuzzy c-means clustering analysis revealed that four distinct clusters of temporal patterns were observed in the NH and HS groups, indicating the kinetic characteristics of the different metabolites (Figure 3A). We then classified the metabolites and found that fatty acyls, organooxygen compounds, and prenol lipids were clustered in the NH and HS groups (Figure 3B). Pathway analyses of metabolites from different clusters revealed that clusters 1–4 in the NH group were mainly enriched in tyrosine and tryptophan biosynthesis, the oxytocin signaling pathway, and GnRH secretion (*p <* 0.05; Figure 3C). Clusters 1–4 in the HS group were mainly enriched in apoptosis, circadian entrainment, gap junction, steroid biosynthesis, and the cortisol synthesis and secretion signaling pathways (*p <* 0.05).

**Figure 4:**
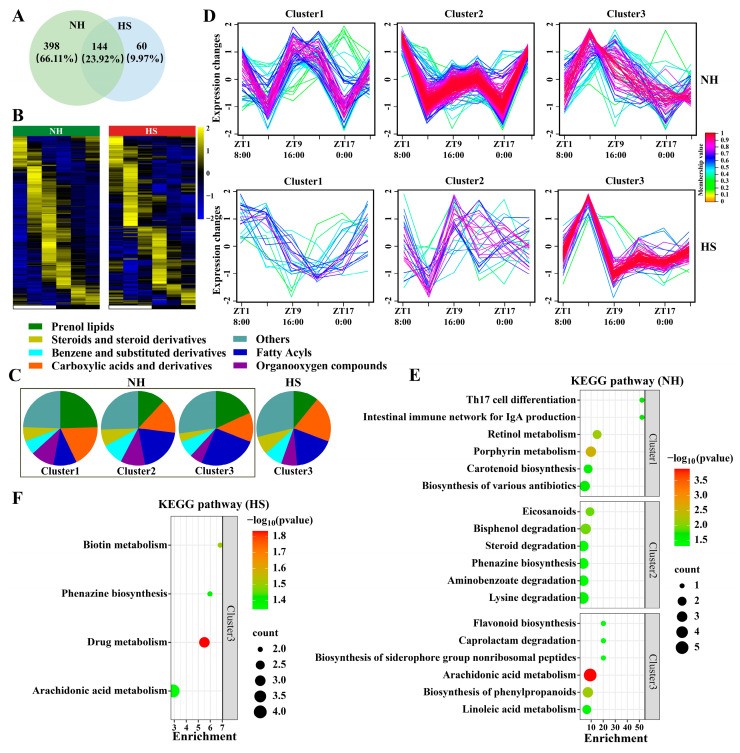
Heat stress alters the circadian rhythm of rumen metabolites in rams. (**A**) Venn diagram showing the number of rhythmic metabolites and also significant differences between groups. (**B**) Heat map showing oscillating metabolites in the NH (**left**) or HS (**right**) groups. (**C**) Classification of metabolites. (**D**) Identification of distinct temporal patterns of metabolites in the NH and HS groups by fuzzy c-means clustering. (**E**,**F**) Pathways annotated for rumen metabolites based on the KEGG database (*p* < 0.05).

To further reveal the role of rumen metabolites in regulating physiological processes in sheep, a JTK analysis of metabolites with significant differences between groups was carried out, and the rhythmic metabolites of NH and HS groups were found to be 66.11% and 9.97% (ADJ.P < 0.05), respectively (Figure 4A). The heat map shows that the peak of rhythmic metabolites mainly appeared in the daytime in the NH group, whereas it was distributed throughout the day in the HS group (Figure 4B). Among these, the abundance of prenol lipids, fatty acyls, and carboxylic acids and their derivatives varied significantly (Figure 4C). Similarly, we applied fuzzy c-means clustering to analyze the different rhythmic metabolites between the NH and HS groups and found that the NH and HS groups had three different temporal pattern clusters (Figure 4D). We then performed a KEGG analysis of metabolites from different clusters and found that NH groups 1–3 were mainly enriched in pathways related to the sperm’s biological functions, such as retinol, arachidonic acid, and linoleic acid metabolism (*p <* 0.05; Figure 4E). However, only cluster 3 was enriched in the biotin metabolism and phenazine biosynthesis in the HS group (*p <* 0.05; Figure 4F). 

### 2.4. HS Alters the Composition of Metabolites in Rumen Fluid and Seminal Plasma of Sheep

**Figure 5:**
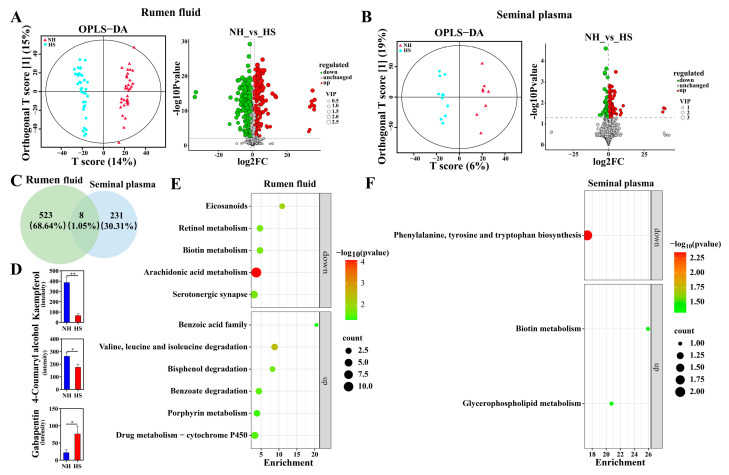
Heat stress alters the composition of metabolites in rumen fluid and seminal plasma. (**A**,**B**) Heat stress altered rumen and seminal plasma metabolomic profiles based on orthogonal partial least-squares discriminant analysis (OPLS-DA) in the NH and HS groups, respectively. Differentially abundant rumen and seminal plasma metabolites were visualized using volcano plots in the NH and HS groups, respectively. (**C**) Venn diagram showing the number of metabolites between rumen and seminal plasma. (**D**) Common differential metabolite in rumen fluid and seminal plasma. (**E**,**F**) Pathways annotated for rumen fluid and seminal plasma metabolites based on the KEGG database (*p* < 0.05). Asterisks indicate significance at *p* < 0.05 (*), *p* < 0.01 (**).

To further reveal the effects of HS on rumen metabolites and sperm biological functions, we analyzed the differential metabolites in rumen fluid and seminal plasma. Our results revealed a clear difference between the metabolic profiles in the NH and HS groups (Figure 5A,B). Analysis of the NH vs. HS comparison showed that the abundances of 213 and 187 metabolites increased, while the abundances of 318 and 52 metabolites decreased in rumen fluid and seminal plasma, respectively (*p <* 0.05; Figure 5A,B). Moreover, we found that specific differential metabolites accounted for 68.64% and 30.31% of the metabolites in the rumen fluid and seminal plasma, respectively (Figure 5C). Among these metabolites, the levels of kaempferol and 4-coumaryl alcohol in rumen fluid and seminal plasma decreased in the HS group, whereas the level of gabapentin increased in the HS group (*p <* 0.05) compared with that in the NH group (Figure 5D). Subsequently, KEGG analysis of the differential metabolites in rumen fluid showed that increased levels of metabolites were enriched for bisphenol degradation and porphyrin metabolism, whereas decreased levels of metabolites were enriched for retinol, arachidonic acid, and biotin metabolism in the HS group (*p <* 0.05; Figure 5E). Compared with rumen fluid metabolites, the HS group was associated with an increase in the levels of seminal plasma metabolites that were enriched in biotin and glycerophospholipid metabolism, but a decrease in the levels of metabolites enriched in phenylalanine, tyrosine, and tryptophan biosynthesis (*p <* 0.05; Figure 5F).

### 2.5. Multi-Omics Integration Analysis of the Effects of HS-Induced Rumen Microbial and Metabolite Changes on Sperm Motility in Rams

**Figure 6:**
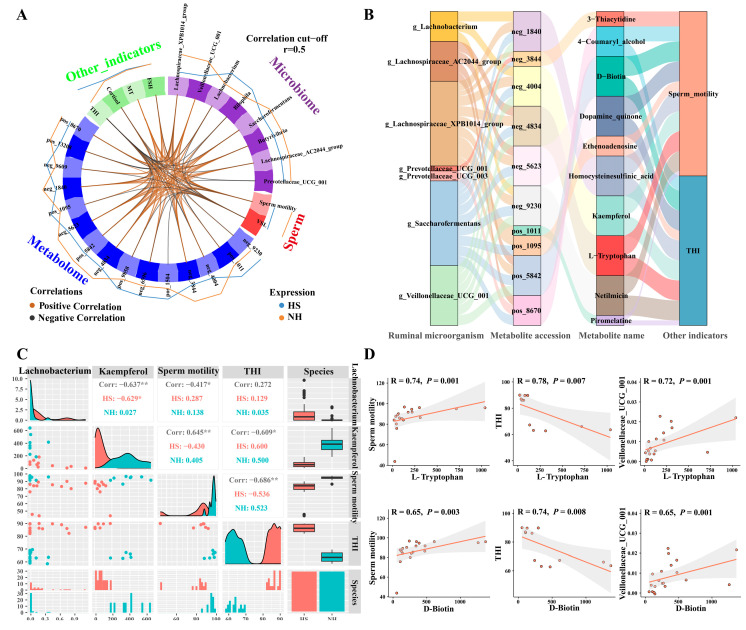
HS altered sperm motility and was related to the diurnal rhythms of rumen microbes and metabolites in rams. (**A**) Circos plot showing variable correlations among microbiome, metabolome, sperm motility parameters, and biochemical indexes. (**B**) Sankey diagram illustrating the complex interactions between microbiome, metabolome, and other indicators’ complex interactions. (**C**,**D**) Spearman correlation between microbiome, metabolome, sperm motility, and THI (r > 0.5, *p* < 0.05). Asterisks indicate significance at *p* < 0.05 (*), *p* < 0.01 (**).

To further analyze the relationship between the changes in rumen microorganisms, metabolites, and sperm motility induced by HS, we analyzed the potential correlations between microbes, metabolites, sperm motility, and other parameters. The results showed that six metabolites (4-coumaryl alcohol, D-biotin, dopamine quinone, homocysteine sulfinic acid, kaempferol, and L-tryptophan) were positively correlated with sperm motility and negatively correlated with the THI (*p <* 0.05; Figure 6A). Three metabolites (ethenoadenosine, netilmicin, and piromelatine) were negatively associated with sperm motility and positively associated with the THI (*p <* 0.05; Figure 6A). Our results further revealed that all of these metabolites were related to rumen microbes, and we mapped a Sankey diagram to describe these complex interactions (Figure 6B). Among these metabolites, kaempferol was negatively correlated with *Lachnobacterium* and positively correlated with *Lachnospiraceae_XPB1014_group*, *Saccharofermentans*, and *Veillonellaceae_UCG_001* (*p <* 0.05; Figure 6B,C). In addition, both L-tryptophan and D-biotin were reduced in the seminal plasma of the HS group (*p <* 0.05). Correlation analysis showed that these metabolites were positively correlated with *Veillonellaceae_UCG_001* and sperm motility, but negatively correlated with the THI (*p <* 0.05; Figure 6D). 

## 3. Discussion

Current research has shown that HS-induced dysbiosis of the gut microbiota impairs spermatogenesis in static models [28]. However, little is known about whether HS damages ram sperm motility by disrupting microbial and metabolite rhythmicity on the circadian time scale. In this study, we elucidated the correlation between HS-induced dysregulation of microbial and metabolite rhythm and low sperm motility; a multi-omics analysis revealed that the rhythm disorder of microorganisms and metabolites may be associated with cortisol, testosterone, LPS, and antioxidant disorders, thus leading to a decrease in sperm motility. To the best of our knowledge, this is the first study to report that HS-induced circadian rhythm disruption in rumen microorganisms and metabolites is closely related to a decrease in sperm motility. 

Recent studies have found that diurnal variations in environmental temperature can synchronize the rhythm of animal body temperature, especially during periods of high air temperature [12,13]. Consistent with previous studies, HS changed the circadian rhythm of body temperature, resulting in a loss of diurnal differences in body temperature. The core body temperature and respiration rate indicate whether animals are experiencing HS [29,30]. In this study, HS increased the core body temperature and respiration rate during the daytime and nighttime. Moreover, when the environment’s THI exceeds 72, rams experience HS [31]. The average THI in the HS group was 86.34 during the experiment; therefore, these results confirmed that rams were in a state of HS. 

Cortisol is an important indicator of stress in rams [32,33]. 17α-Hydroxypregnenolone, a precursor for cortisol synthesis, showed rhythmic changes in the HS group, and metabolome analysis found it enriched in cortisol synthesis and secretion signaling pathways. To further confirm this finding, analysis of the cortisol concentration found that HS increased serum cortisol levels in rams. Stress-induced elevated cortisol concentrations inhibited testosterone synthesis in Sertoli cells. Testosterone is an important reproductive hormone that promotes spermatogenesis, maturation, and motility through the androgen receptor pathway; a reduction in its concentration can impede spermatogenesis and decrease sperm viability, leading to reproductive disorders in male animals [34,35]. Consistent with the results of previous studies, we found that elevated cortisol levels in the HS group reduced testosterone levels at zeitgeber time 9 (the highest THI and body temperature point) and significantly reduced sperm motility in sheep. These results suggest that HS interferes with sperm motility by affecting cortisol and testosterone synthesis in rams; however, the mechanism by which HS affects the syntheses of cortisol and testosterone requires further research. 

An increasing number of studies have shown that gut microbes play a key role in spermatogenesis and hormone synthesis. When the external environment disturbs microorganisms, it affects the synthesis of hormones such as cortisol and testosterone and thus causes reproductive dysfunction in the host [36,37,38]. Studies have found that *Lachnospiraceae*, which resides in the gut and urogenital tract, inactivates cortisol by cutting the side chain after synthesizing steroid-17,20-desmolase, and when the abundance of *Lachnospiraceae* is reduced, the cortisol response is intensified [39]. These results suggest that HS decreased the abundance of *Lachnospiraceae*, which may have enhanced the inhibitory effects of cortisol on testosterone synthesis in Sertoli cells [40]. In addition, a previous study found that *Prevotellaceae* was positively correlated with testosterone synthesis [41]. This study found that HS led to the loss of circadian rhythm and significantly reduced its abundance, which was consistent with the results observed after the decrease in testosterone levels in the HS group. However, how these microbes target and regulate cortisol response and testosterone synthesis requires further study. 

LPS, as a microbe-associated molecular pattern, breaches the intestinal barrier to enter the blood circulation and subsequently reduces the expression of tight junction proteins and gap junction α proteins in Sertoli cells via Toll-like receptor-mediated signaling pathways, thus destroying the blood–testis barrier and eventually leading to spermatogenic cell apoptosis and sperm DNA damage [42]. This found that the serum LPS concentration increased significantly in the HS group. These results suggest that HS may destroy the blood–testis barrier via LPS. Moreover, we analyzed the differential metabolites in seminal plasma and found that the sphingosine was enriched in the apoptosis signaling pathways. A previous study revealed that sphingosine was negatively correlated with *Lachnospiraceae*, which plays an important role in blocking cell proliferation and promoting apoptosis after passing through the blood–testis barrier, blockings the transition of round spermatozoa from the S2 to S3 phase [43]. Therefore, these results suggest that first, HS may destroy the blood–testis barrier through LPS, and second, the sphingosine-induced apoptosis of spermatogenic cells in the testes leads to the inhibition of spermatogenesis and a decrease in sperm motility in rams. However, microbe-derived LPS not only disrupts the blood–testis barrier, but also causes an imbalance in testicular redox reactions and intrinsic antioxidant mechanisms, ultimately leading to an increase in the reactive oxygen species (ROS). When the ROS concentration exceeds the physiological range, it causes Leydig cell dysfunction, sperm DNA damage, and sperm motility decline [44]. Tryptophan is an important multifunctional molecule in enterochromaffin cells mediated by *Clostridium* spp., which can enhance barrier function and mitigate oxidative stress by activating the AhR-Nrf2 crosstalk pathway [45]. In this study, we found that the levels of L-tryptophan, which is enriched in the tryptophan biosynthesis signaling pathway, was significantly reduced in the seminal plasma of the HS group. Correlation analysis showed that this molecule was positively correlated with sperm motility and *Veillonellaceae_UCG_001*, but negatively correlated with the THI. These results suggest that the reduction in tryptophan levels in seminal plasma caused by HS may result in oxidative stress. In addition, kaempferol, a flavonol, has antioxidant and anti-inflammatory properties that can protect against HS-induced Sertoli cell damage and spermatogenesis impairment. Furthermore, it can reduce C-reactive protein levels by increasing macrophage activity, thereby reducing LPS-induced proinflammatory cytokine expression [46]. In line with the results of previous studies, our study found that kaempferol was negatively correlated with *Lachnobacterium* and the THI and positively correlated with *Veillonellaceae_UCG_001* and sperm motility. Thus, these results suggest that HS may reduce the antioxidant capacity of sheep spermatozoa via the modulation of microbe-associated metabolites, leading to decreased sperm motility; however, the exact molecular mechanisms underlying these processes requires further study.

Small molecules produced by gut microbes, including vitamins and bile acids, are important intermediate signaling molecules involved in the regulation of testicular spermatogenesis [47,48]. D-biotin, also known as vitamin B7, plays an important role in maintaining sperm mitochondrial membrane potential stability and protecting sperm from DNA damage [49]. In this study, we found that the level of D-biotin, which was enriched in vitamin digestion and absorption signaling pathways in the HS group, was significantly reduced in seminal plasma; this small molecule was positively correlated with sperm motility and *Veillonellaceae_UCG_001*, but negatively correlated with the THI. These results indicate that HS decreased the abundance of *Veillonellaceae_UCG_001* and D-biotin, which may be an important reason for the decrease in sperm motility in Hu rams. In addition, vitamin A plays a crucial role in male gonadal meiosis after birth, leading to abnormal testicular spermatogenesis when intestinal microbiota-driven bile acid signaling leads to vitamin A metabolic disorders [48]. Among the microbes involved in this process, *Bilophila* can utilize sulfites in taurine as terminal electron acceptors to regulate bile acid metabolism; their increased abundance leads to increased consumption of free taurine in the gut, which further leads to decreased levels of taurine-conjugated bile acids [50]. Consistent with previous results, HS significantly increased the abundance of *Bilophila* and significantly decreased the levels of retinoate, an active metabolite of vitamin A. These results suggest that HS may disrupt spermatogenesis via microbe-mediated vitamin A synthesis. However, further studies are necessary to reveal the potential mechanism of microbe metabolites-induced spermatogenesis disorder based on rumen bacteria transplantation tests.

## 4. Materials and Methods

### 4.1. Animal Experiments and Sample Collection

All animal experimental procedures were approved by the Animal Care and Use Committee of Anhui Agricultural University (AHAUB2022008). This study was conducted at Haiqinsheng Eco-Farming Co., Ltd., in Dingyuan, China (Latitude: 32°34′ N; Longitude: 117°29′ E; Altitude: 84 m above sea level) from May to July, 2022. The temperature in the sheep houses were recorded using an automatic temperature determinator (Qingzheng, Beijing, China); the average environmental temperatures were 18.71 ± 2.86 °C and 33.58 ± 3.57 °C in May and July, respectively. The prevalent photoperiod was 13 h daylight and 11 h darkness. In this study, two-year-old Hu rams (67.23 ± 2.56) with year-round estrous were selected, and semen samples were collected from the six healthy Hu rams with an artificial vagina twice a week. Semen quality and sperm motility were selected for the follow-up experiment and evaluated using a computer-assisted sperm analyzer (CASA, sperm class analyzer-5.4.0.0; MICROPTIC Supply, Barcelona, Spain). During the experiment, all sheep were fed twice daily in the morning and evening, and had ad libitum access to fresh water. Sheep diets were formulated to meet the nutrient requirements for breeding. The composition and nutrition levels of the diets are shown in Table S1. Respiratory rate (RR) was measured using a stethoscope at 4 h intervals for 24 h. The relative humidity in the sheep houses was recorded using a humidity determinator (Qingzheng, Beijing, China), and the temperature–humidity index (THI) was calculated according to previous reports [31]. When the THI was <72, sheep were in a non-heat stress (NH) state, and when the THI was >72, they were in a heat stress (HS) state.

### 4.2. Ram Sampling of Semen, Blood, and Ruminal Content

Semen was collected weekly under NH and HS conditions using an artificial vaginal technique to assess semen quality [51]. The remaining semen specimens were separated from spermatozoa and seminal plasma using centrifugation (10,000× *g*, 4 °C, 10 min). The separated seminal plasma samples were immediately frozen in liquid nitrogen and used for subsequent testing. In addition, diurnal changes in rectal temperature were measured with a digital thermometer (Delta Track, Pleasanton, CA, USA) at 4 h intervals throughout the day at the end of the last evaluation of semen quality under NH and HS conditions. Blood samples were collected using jugular vein puncture and were centrifuged (4 °C, 3000× *g*, 10 min) to obtain serum. Simultaneously, rumen contents were collected using a gastric tube immediately after blood collection [52]. Briefly, a gastric tube rumen fluid sampler (Colibri Pastoral, Wuhan, China) was inserted to a depth of approximately 100 cm until the probe tip reached the rumen ventral sac. A large-capacity sterile syringe (200 mL) was connected to the rear of the sampling tube, and rumen contents were drawn out using the syringe. The collected contents were filtered through four layers of sterile gauze, and the filtered rumen fluid was collected in sterile 50 mL centrifuge tubes and immediately frozen in liquid nitrogen for DNA extraction and subsequent microbial and metabolite analyses.

### 4.3. Ruminal Microbial DNA Extraction and 16S rRNA Sequencing

Total genomic DNA was extracted from rumen fluid using a TGuide S96 Fecal Genomic DNA Extraction Kit (Tiangen, Beijing, China) according to the manufacturer’s protocol. The full-length of 16S rRNA (V1–V9) was amplified using specific primers (27F: 5′-AGRGTTTGATYNTGGCTCAG-3′ and 1492R: 5′-TASGGHTACCTTGTTASGACTT-3′). The amplicon library was subjected to high-throughput sequencing on a PacBio Sequel II platform (Pacific Biosciences, Menlo Park, CA, USA). Amplicon sequence variants (ASVs) were generated after denoising using DADA2 in QIIME2 (version 2020.06). Taxonomic annotation of ASVs was performed using the SILVA 138 database. ASVs that did not pass the 0.005% filter of the total number of sequences were also excluded. Alpha and beta diversity indices were analyzed using the QIIME2 software (v 1.9.1) in BMK Cloud (www.biocloud.net).

### 4.4. Metabolomic Analysis of Rumen Liquid and Seminal Plasma

Rumen liquid and seminal plasma samples (100 μL) were mixed with 500 µL of methanol and acetonitrile (volume ratio of 1:1), respectively, vortexed for 30 s, and sonicated for 10 min. All samples were left for 1 h (−20 °C) and then centrifuged (4 °C, 10,000× *g*, 15 min); 500 µL of the supernatant was transferred to an EP tube, to which 160 µL of acetonitrile and water (volume ratio of 1:1) was added, and then vortexed for 30 s. All samples were centrifuged again, and 120 µL of supernatant was transferred into a 2 mL sample bottle. Finally, 10 µL of the solution from each sample bottle was used for testing.

The liquid chromatography-mass spectrometry system for metabolomics analysis was composed of a Waters Acquity I-Class PLUS ultra-high performance liquid tandem Waters Xevo G2-XS QT high-resolution mass spectrometer (Beijing Biomarker Biotechnology Company, Beijing, China). Raw data were collected using MassLynx V4.2 and further processed using Progenesis QI software (version 4.0). The online METLIN database and Biomark’s self-built library were used for identification based on Progenesis QI software, and the mass deviation of fragment ion identification was within 100 ppm. Raw peak area information was normalized using the total peak area normalization method. Differential metabolites were identified based on fold change (FC) > 2.0, *p* < 0.05; and variable importance in projection (VIP) > 1 in rumen liquid, and differential metabolites in seminal plasma were identified based on FC > 1.5, *p* < 0.05, and VIP > 1. Orthogonal partial least-squares discriminant analysis (OPLS-DA) and Kyoto Encyclopedia of Genes and Genomes (KEGG) pathway enrichment were analyzed using the BMK Cloud (www.biocloud.net).

### 4.5. Biochemical, Vitamin, and Endocrine Serum Quantifications

Serum concentrations of lipopolysaccharide (LPS), Vitamin D3 (Vit D3), testosterone, LH, FSH, melatonin (MT), and cortisol were determined using a commercially available ELISA kit (Jianglai, Shanghai, China) following the manufacturer’s instructions. 

### 4.6. Statistical Analysis

The non-parametric Jonckheere–Terpstra–Kendall (JTK) Cycle (in R software, version 4.3.1) was used to analyze the significance and amplitude of 24 h rhythms, as previously described [53]. The Wilcoxon rank-sum test was used to analyze the differences in non-parametric data between the two groups. For biochemical index data, Student’s t-test was used to compare the differences between two groups, and the differences in biochemical indices between day and night were analyzed using repeated-measures analysis of variance with Bonferroni’s comparison. Statistical analyses were performed using SPSS software (version 20.0; SPSS Inc., Chicago, IL, USA). In addition, different omics datasets were analyzed using the mixOmics package in R [54]. The correlations among the microbes, metabolites, sperm motility, and other parameters were analyzed using the Hmisc package in R. Rhythmic microorganisms and metabolites were grouped into different clusters using the fuzzy c-means algorithm implemented in R package Mfuzz [55]. Figures were generated using Prism software (version 7.0; GraphPad Software, Inc., La Jolla, CA, USA).

## 5. Conclusions

This study reveals that HS decreased sperm motility in Hu rams. Serum indicators found that HS increased the concentrations of cortisol and LPS and decreased the testosterone concentration. At the rumen microorganism level, HS decreased the abundance of *Lachnobacterium* and *Lachnospiraceae* and disrupted the circadian rhythms of *Veillonellaceae_UCG_001* and *Prevotellaceae*. At the metabolite level, HS altered the circadian rhythm and composition of metabolites, and downregulated metabolites such as kaempferol and L-tryptophan in rumen fluid and seminal plasma. Multi-omics integration analysis further confirmed that HS-induced circadian rhythm disruption in rumen microorganisms and metabolites was closely related to a decrease in sperm motility. Therefore, our findings provide a new perspective for HS-induced sperm motility decline in sheep through the gut–testis axis on a diurnal timescale.

## Data Availability

All data generated or analyzed during this study are included in this article, and materials are available from the authors upon reasonable request.

## Supplementary Materials

The following supporting information can be downloaded at: https://www.mdpi.com/article/10.3390/ijms252011161/s1.
