# Supplementary material for: Environmental Heat Stress Decreases Sperm Motility by Disrupting the Diurnal Rhythms of Rumen Microbes and Metabolites in Hu Rams
Source: Int J Mol Sci. 2024 Oct 17;25(20):11161. doi: 10.3390/ijms252011161 (PMC11508439; doi:10.3390/ijms252011161)
Supplement: Supplementary file 1 [file ijms-25-11161-s001.zip › ijms-3177176-supplementary.pdf]

Supplementary Materials

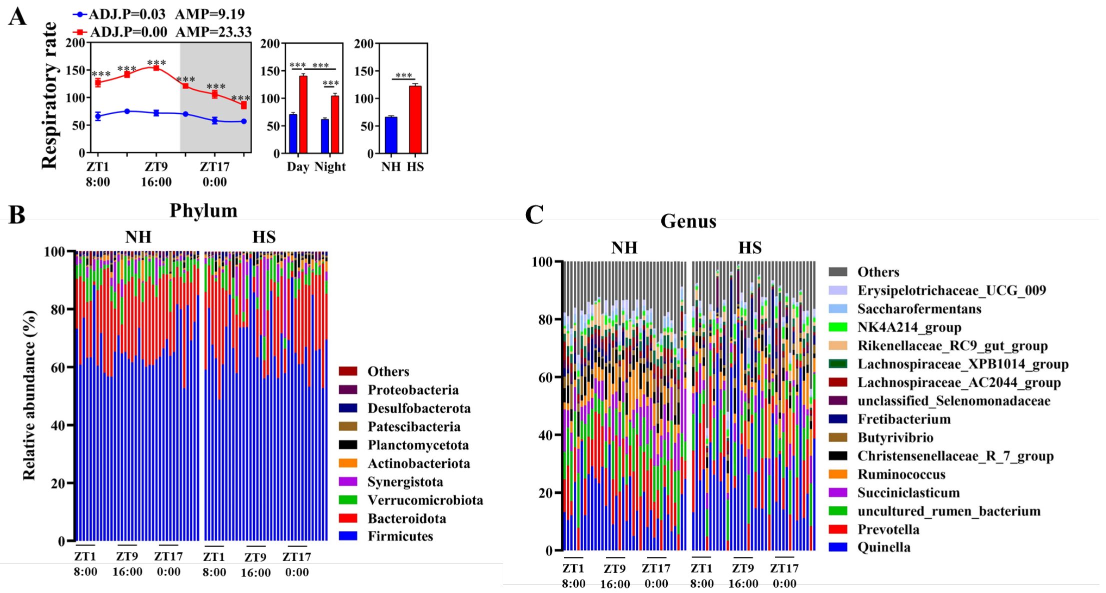

Figure S1. Heat stress affects respiration rate and the composition of rumen microbes in rams. (A) The diurnal rhythms based on Jonckheere−Terpstra−Kendall (JTK) analysis of respiration rate. (B) Taxonomic composition of microbiota at the phylum level. (C) Taxonomic composition of microbiota at the genus level. Asterisks indicate significance at $p < 0.05$ (*), $p < 0.01$ (**), $p < 0.001$ (***).

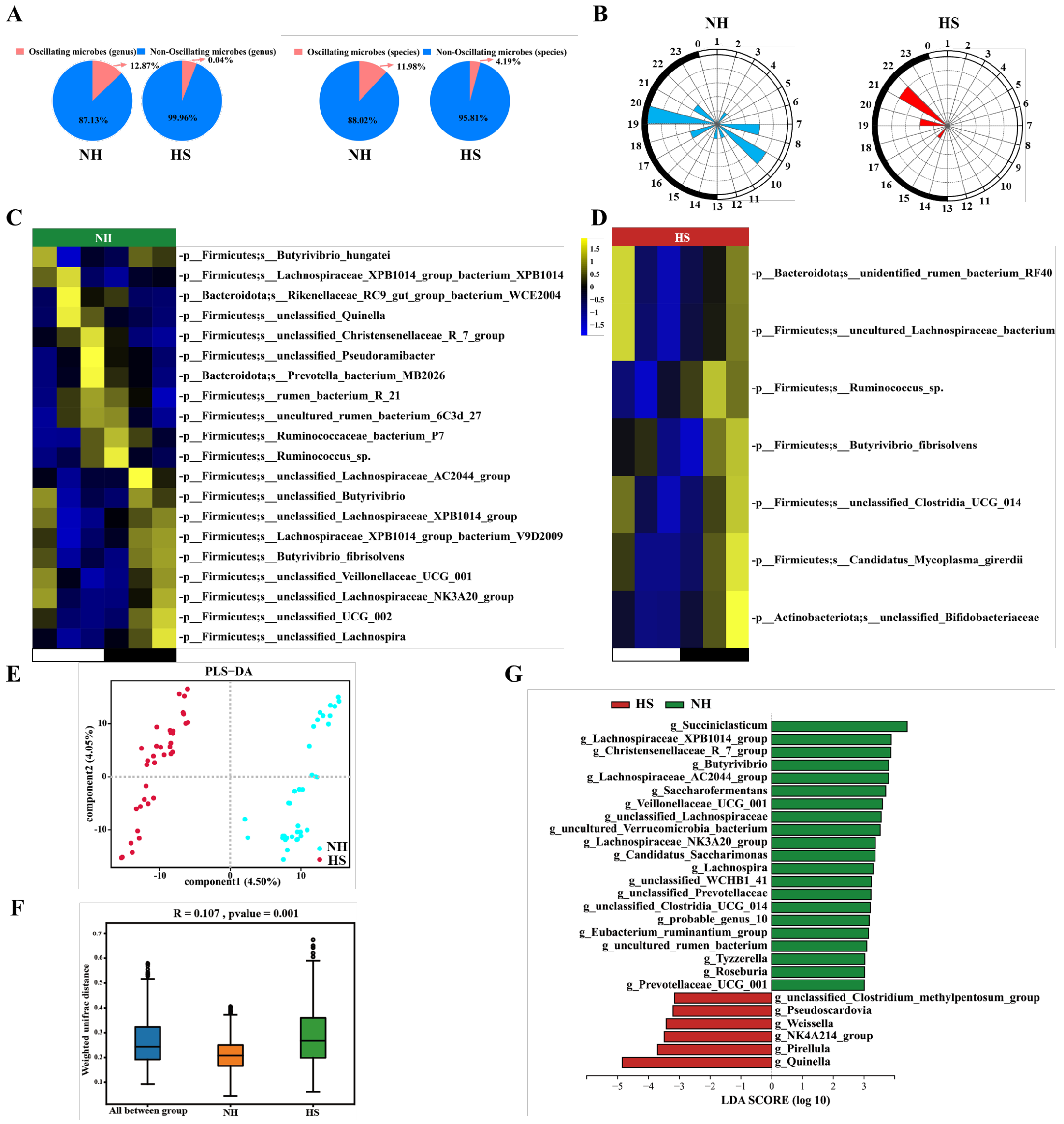

Figure S2. HS alters the circadian rhythm and composition of rumen microbes in rams. (A) Pie chart showing the percentage of oscillating and non-oscillating microbes in the NH and HS groups. (B) The polar plot represent time when the microbes (species) peak level of abundance appeared, blue (NH group) or red (HS group) shading represents the number of rhythmic microbes with an estimated peak value for each time as determined by JTK analysis. The radius of black concentric circles indicates the number of rhythmic microbes, and the minimum radius of the black concentric circle represents one microbe. The black arc on the left side of the polar plot indicates the day/night cycle.

(C-D) Heat map showing oscillating microbes (species) in the NH (left) or HS (right) groups. (E) Analysis of gut microbiota using OPLS-DA. (F) ANOSIM analysis of ASV levels using weighted UniFrac. (G) LEfSe analysis at the genus level (LDA > 3). ANOSIM, analysis of similarities; LEfSe, linear discriminant analysis effect size; LDA, linear discriminant analysis.

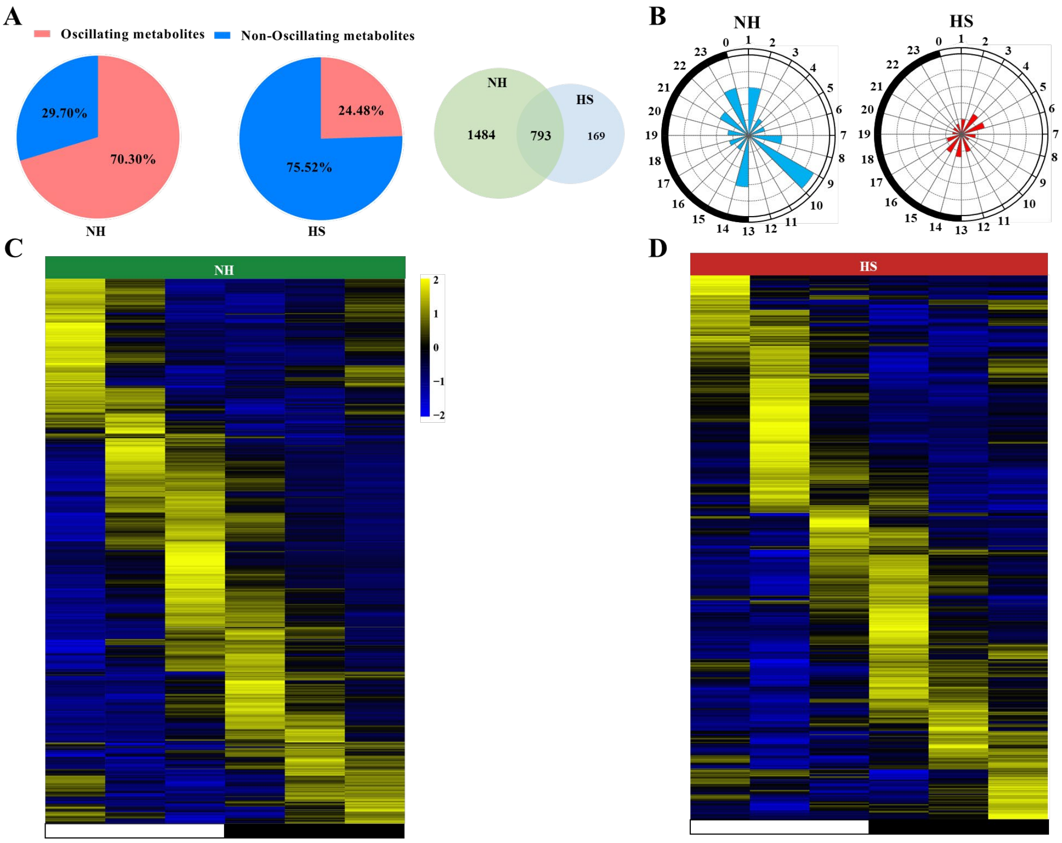

Figure S3. Heat stress alters the circadian rhythm of rumen metabolites in rams. (A) Pie chart and venn diagram showing the percentage of oscillating and non-oscillating metabolites in the NH and HS groups. (B) The polar plot represent time when the microbes (species) peak level of abundance appeared, blue (NH group) or red (HS group) shading represents the number of rhythmic metabolites with an estimated peak value for each time as determined by JTK analysis. The radius of black concentric circles indicates the number of rhythmic metabolites, and the minimum radius of the

black concentric circle represents eighty metabolites. The black arc on the left side of the polar plot indicates the day/night cycle. (C-D) Heat map showing oscillating metabolites (species) in the NH (left) or HS (right) groups.

Table S1. Composition of the basal diets

Items	Contents
Ingredients (%)	
Peanut seedling	49.5
Maize	26.0
Wheat bran	9.0
Soybean meal	14.0
$NaHCO_3$	0.5
NaCl	0.5
Premix[1]	0.5
Total	100
Nutrient levels	
Digestible energy (MJ/kg)	18.14
Crude protein	16.35
Neutral detergent fiber	46.17
Acid detergent fiber	27.84
Calcium	0.67
Phosphorus	0.45

[1]Note: Premix provided the following per kg of feed: vitamin A 13,000 IU, vitamin D 4,000 IU, vitamin E 200 IU, Cu 15 mg, Fe 65 mg, Mn 60 mg, Zn 55 mg, I 1.5 mg, Se 0.85 mg, Co 0.4 mg.
